# Assessment of the Quality of Reporting of Randomised Controlled Trials in Otorhinolaryngologic Literature – Adherence to the CONSORT Statement

**DOI:** 10.1371/journal.pone.0122328

**Published:** 2015-03-20

**Authors:** Jeroen P. M. Peters, Lotty Hooft, Wilko Grolman, Inge Stegeman

**Affiliations:** 1 Department of Otorhinolaryngology and Head & Neck Surgery, University Medical Center Utrecht, Utrecht, The Netherlands; 2 Brain Center Rudolf Magnus, University Medical Center Utrecht, Utrecht, The Netherlands; 3 Dutch Cochrane Centre, Julius Center for Health Sciences and Primary Care, University Medical Center Utrecht, Utrecht, The Netherlands; Liverpool School of Tropical Medicine, UNITED KINGDOM

## Abstract

**Background:**

Randomised Controlled Trials (RCTs) are the preferred study design when comparing therapeutical interventions in medicine. To improve clarity, consistency and transparency of reporting RCTs, the Consolidated Standards of Reporting Trials (CONSORT) statement was developed.

**Objectives:**

(1) To assess the quality of reports and abstracts of RCTs in otorhinolaryngologic literature by using CONSORT checklists, (2) to compare the quality of reports and abstracts of otorhinolaryngologic RCTs between the top 5 general medical journals and top 5 otorhinolaryngologic journals, and (3) to formulate recommendations for authors and editors of otorhinolaryngologic (‘ENT’) journals.

**Methods:**

Based on 2012 ISI Web of Knowledge impact factors, the top 5 general medical and ENT journals were selected. On 25 June 2014, using a highly sensitive Cochrane RCT filter and ENT filter, possibly relevant articles since January 1st, 2010 were retrieved and relevant RCTs were selected. We assessed how many CONSORT items were reported adequately in reports and abstracts and compared the two journal types.

**Results:**

Otorhinolaryngologic RCTs (n = 15) published in general medical journals reported a mean of 92.1% (95% confidence interval: 89.5%–94.7%) of CONSORT items adequately, whereas RCTs (n = 18) published in ENT journals reported a mean of 71.8% (66.7%–76.8%) adequately (p < 0.001). For abstracts, means of 70.0% (63.7%–76.3%) and 32.3% (26.6–38.0%) were found respectively (p < 0.001). Large differences for specific items exist between the two journal types.

**Conclusion:**

The quality of reporting of RCTs in otorhinolaryngologic journals is suboptimal. RCTs published in general medical journals have a higher quality of reporting than RCTs published in ENT journals. We recommend authors to report their trial according to the CONSORT Statement and advise editors to endorse the CONSORT Statement and implement the CONSORT Statement in the editorial process to ensure more adequate reporting of RCTs and their abstracts.

## Introduction

Randomised Controlled Trials (RCTs) are the preferred type of study design when comparing therapeutical interventions in medicine. The study design prevents selection and confounding bias and permits blinding of participants and researchers [1].

However, poor reporting of RCTs can reduce their usefulness [2, 3]. Clinicians and researchers must have access to clear, transparent and complete information to assess the quality and results of a trial accurately. Various aspects of RCTs may lead to bias when reported inadequately. To be able to assess the risk of publication bias and selective reporting, trials are advised to be registered in trial registers before patient enrolment and trial protocols should be published [4, 5]. Finally, adequate reporting of the randomisation procedure, allocation concealment and blinding is essential to value the trial and its results [3, 6, 7].

To improve clarity and transparency of reporting of trials, the Consolidated Standards of Reporting Trials (CONSORT) statement (www.consort-statement.org) was developed in 1996 [8] and revised in 2001 [9] and 2010 [10, 11]. The CONSORT Statement and the corresponding checklist summarize all essential items that should be reported in an RCT. Several extensions have been published since, i.e. CONSORT for Abstracts [12], CONSORT for harms [13] and CONSORT for non-pharmacological treatments [14]. Journal endorsement of the CONSORT Statement may beneficially influence the completeness of reporting of trials [15–17].

The objective of this study was to assess the quality of reports and abstracts of RCTs in otorhinolaryngologic literature. Therefore, we scored articles in the otorhinolaryngologic research field with CONSORT checklists. Secondary, we compared the quality of reporting RCTs between the top 5 general medical journals and the top 5 otorhinolaryngologic journals. Finally, we aim to formulate recommendations for authors and editors of otorhinolaryngologic journals.

## Methods

### Journals

We selected the top 5 general medical journals and top 5 otorhinolaryngologic journals (‘ENT journals’, Ear Nose Throat [ENT]), based on their 2012 ISI Web of Knowledge impact factors (www.webofknowledge.com, date of access June 25^th^, 2014). The journals and impact factors are shown in Table 1. The top 5 journals in general medical literature are *New England Journal of Medicine* (NEJM), followed by *The Lancet* (Lancet), *Journal of the American Medical Association* (JAMA), *British Medical Journal* (BMJ) and *PLOS Medicine* (PLOS Med). In ENT literature, *Ear & Hearing* (Ear Hear) is the journal with the highest impact factor, followed by *Journal of the Association for Research in Otolaryngology* (JARO), *Head & Neck* (Head Neck), *Hearing Research* (Hear Res) and A*udiology & Neurotology* (Audiol Neurotol).

**Table 1:** Impact Factors 2012 Top 5 general medical and ENT journals.

Journal	Impact Factor*
General medical journals	
1. New England Journal of Medicine (NEJM)	51.658
2. Lancet	39.060
3. Journal of the American Medical Association (JAMA)	29.978
4. British Medical Journal (BMJ)	17.215
5. PLOS Medicine (PLOS Med)	15.253
ENT journals	
1. Ear & Hearing (Ear Hear)	3.262
2. Journal of the Association for Research in Otolaryngoloy (JARO)	2.952
3. Head & Neck (Head Neck)	2.833
4. Hearing Research (Hear Res)	2.537
5. Audiology & Neurotology (Audiol Neurotol)	2.318

* Source: ISI Web of Knowledge 2012, Journal Citations Reports (JCR) via www.webofknowledge.com, accessed on June 25^th^, 2014.

ENT = Ear Nose Throat.

### Search

**Fig 1:**
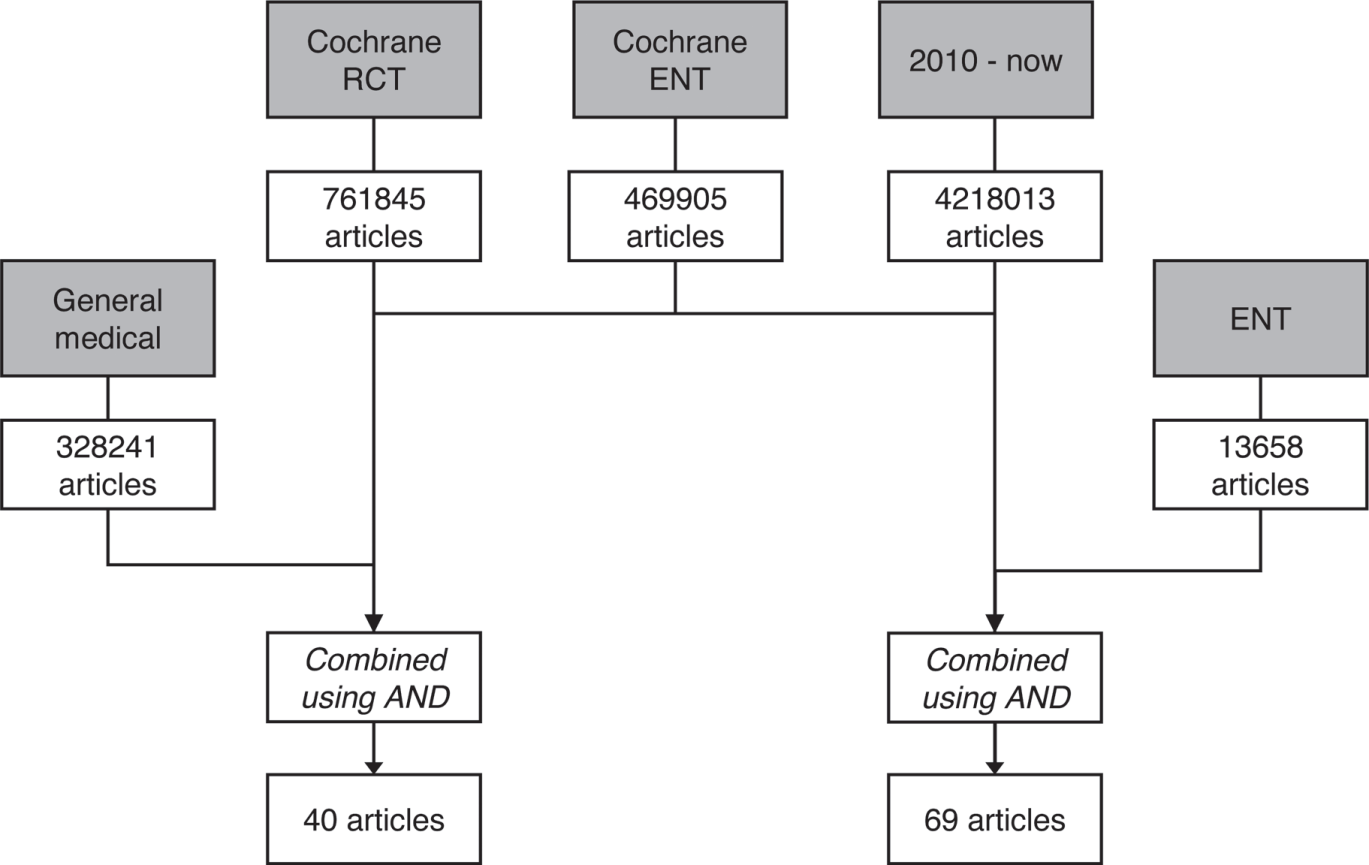
Flowchart of search. Date of search: June 25^th^, 2014. For complete search syntaxes: see S1 File.

We searched Pubmed on June 25^th^ 2014 for relevant literature using two filters developed and tested by the Cochrane Collaboration. First, an adapted version of the ENT search filter was used to identify otorhinolaryngologic articles (S1 File) [18]. Second, to retrieve only RCTs, the highly sensitive RCT filter was used [19]. A date restriction was applied to yield only articles published since January 1^st^, 2010 (for complete search: see S1 File). Finally, a combination was made with a search syntax for the top 5 general medical journals and the top 5 ENT journals respectively using Boolean operator AND (Fig. 1).

### Study selection

Two authors (JPMP and IS) independently assessed titles and abstracts of the retrieved articles to check if the study was indeed conducted in the otorhinolaryngologic field and if it was an RCT. To be considered as a study in the otorhinolaryngologic field, studies must assess patients generally treated by otorhinolaryngologists or investigate a procedure generally performed by otorhinolaryngologists, including head and neck surgery (S1 File). To be considered an RCT, studies must have randomised their human population in two or more groups receiving a therapeutic intervention. Secondary analysis of previously reported RCTs or economic evaluations were excluded. Discrepancies between the two independent reviewers were discussed until consensus was reached.

### CONSORT 2010 adherence

To score the quality of reporting, the most recent version of the CONSORT Statement was used (CONSORT 2010, www.consort-statement.org) [10]. If studies assessed a non-pharmacological treatment, the additions stated in the CONSORT Statement for non-pharmacological interventions were taken into account and scored according to the descriptions in this extension [14].

The included articles were read full text independently by two authors (JPMP and IS) and differences in opinion were discussed until consensus was reached. We evaluated the number of items of the CONSORT 2010 checklist that were adequately reported. A more detailed explanation on how items were assessed can be found in S2 File. Next, we compared reporting of specific items between the general medical journals and ENT journals.

When scoring item 19 (Harms), we took the additions for reporting harms as explained in the CONSORT extension for reporting harms into account [13].

### CONSORT for Abstracts adherence

We scored item 1b (Structured summary) separately on a checklist specifically designed to assess abstracts, published along with the CONSORT for Abstracts extension [12]. A more detailed explanation on how all 16 items were assessed can be found in S3 File. Again, we also compared the adequate reporting of items per journal type.

Furthermore, we hypothesised that abstracts with more words would report more items adequately. To test this hypothesis, we counted the words of the abstract excluding title, authors names, affiliations, journal and volumes numbers and key words. These counts are shown with standard deviations and range.

### Data analysis

In total, there are 37 CONSORT (sub)items. Item 1b (Structured summary) is scored separately. The number of adequately reported items was divided by a possible total of 36, resulting in a percentage. The higher the percentage, the more adequately authors reported their trial. For the assessment of abstracts, the number of adequately reported items was divided by a possible total of 16, because one of the items on the checklist is specific to conference abstracts only (name of authors), and therefore not scored.

Means, medians and 95% confidence intervals (CI) were calculated. The 2-tailed Mann Whitney U test for 2 independent samples was used to compare CONSORT scores and CONSORT for Abstracts scores for articles published in general medical journals with ENT journals. The correlation between adequate reporting of CONSORT for Abstracts items and number of words was calculated using Spearman’s rho. Statistical tests were performed using SPSS v20 statistics package. A *p*-value of < 0.05 was considered statistically significant.

## Results

### Search

The search process is showed in Fig. 1. The combined search syntaxes yielded 40 articles in the general medical journals and 69 articles in the ENT journals.

### Study selection

Of the 40 articles in the general medical journals, 3 articles were neither considered otorhinolaryngologic research nor an RCT and were therefore excluded. Seventeen RCTs were not considered otorhinolaryngologic and finally 17 otorhinolaryngologic studies were no RCTs. We included the remaining 15 RCTs in otorhinolaryngology (Table 2).

Of the 69 articles in the ENT journals, 1 article was excluded because it was neither an RCT nor conducted in otorhinolaryngology. We excluded 5 RCTs not performed in otorhinolaryngology and excluded 45 non-RCT studies in otorhinolaryngology. This large number of exclusions can be explained by phrases like ‘stimuli were presented randomly’ or ‘in random order’ that were detected by our highly sensitive RCT filter. In the end, we included 18 RCTs on otorhinolaryngologic topics (Table 3).

**Table 2:** Assessment of retrieved articles in general medical journals.

		ENT?		
		yes	no	
**RCT?**	yes	15	17	**32**
	no	5	3	**8**
		**20**	**20**	***40***

ENT = Ear Nose Throat, RCT = Randomised Controlled Trial

**Table 3:** Assessment of retrieved articles in ENT journals.

		ENT?		
		yes	no	
**RCT?**	yes	18	5	**23**
	no	45	1	**46**
		**63**	**6**	***69***

ENT = Ear Nose Throat, RCT = Randomised Controlled Trial

### CONSORT 2010 adherence

All 15 articles published in general medical journals (NEJM 4, Lancet 1, JAMA 7, BMJ 3, PLOS Med 0) reported a mean of 92.1% (95% CI: 89.5%–94.7%; median 91.9%) of CONSORT items adequately. All 18 articles published in ENT journals (Ear Hear 2, JARO 1, Head Neck 7, Hear Res 2, Audiol Neurotol 6) reported a mean of 71.8% (66.7%–76.8%; median 74.3%) of CONSORT items adequately. Importantly, between these two journal types, there is a statistically significant difference in adequately reported CONSORT 2010 items (Mann Whitney U, *p* < 0.001).

**Fig 2:**
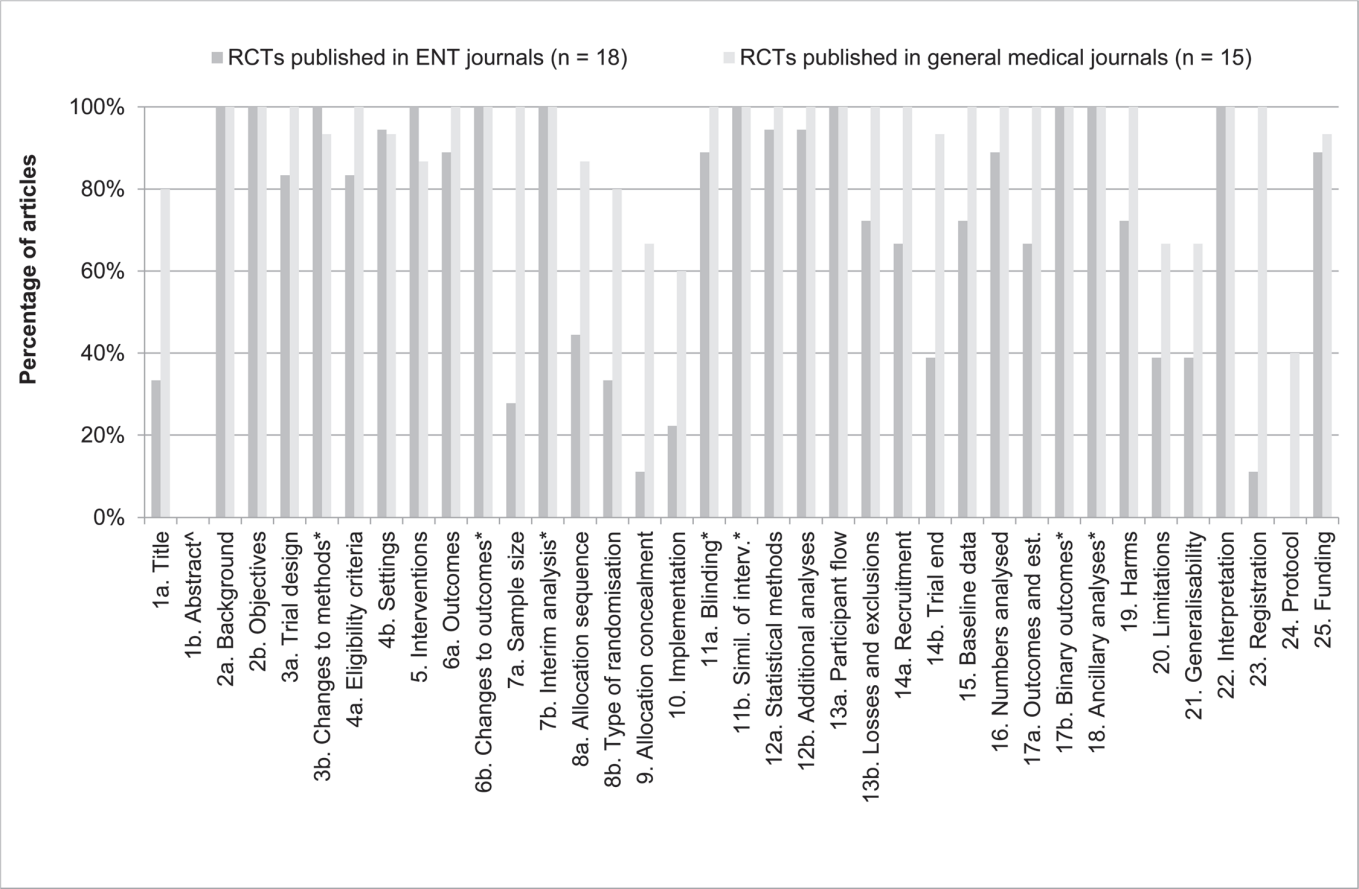
Reporting of CONSORT items per journal type. The percentage of articles reporting CONSORT items adequately (in %), sorted per journal type (articles published in general medical journals, n = 15; articles published in ENT journals, n = 18), using the CONSORT 2010 checklist [10]. ^ Item 1b: item Structured summary is assessed with the specific CONSORT for Abstracts checklist [12], see Fig. 3. * Marked items concern optional items. When possible in the study and adequately reported, the item was scored as ‘adequately reported’. When possible, but not reported, the item was scored as ‘inadequately reported’. If not possible, the item was not scored as ‘inadequately reported’, but left open.

We also compared the difference in reporting individual items between the general medical and ENT journals. Fig. 2 shows how many articles reported individual CONSORT items adequately, sorted per journal type. There are several striking differences to be observed. First, the authors of two out of three RCTs in ENT journals do not mention that they conducted an RCT in the title of their manuscript. Even though the authors of one in five RCTs in general medical journals also not state this, the number is much higher for articles published in ENT journals. Second, articles in ENT journals hardly state sample size calculations (28%, item 7a) and subsequently inadequately reported why the trial was ended (39%, item 14b). Third, both articles in general medical journals and in ENT journals report Randomisation items (items 8a and 8b, 9, 10; Sequence generation, Allocation concealment mechanism, Implementation, respectively) inadequately; however, articles in ENT journals reported worse (44%, 33%, 11%, 22% respectively). Fourth, items 20 and 21 (Limitations and Generalisability, respectively) are adequately reported in only ∼40% of articles in ENT journals. Fifth, only 11% of articles published in ENT journals state the name of trial registry and the registration number, whereas this is 100% in general medical journals. Lastly, not one article in ENT journals stated where the full trial protocol could be accessed (40% for general medical journals).

### CONSORT for Abstracts adherence

Item 1b (Structured summary) is not reported in Fig. 2, because we assessed the reporting of abstracts with the CONSORT for Abstracts checklist. Articles published in general medical journals report a mean of 70.0% (63.7%–76.3%; median 68.6%) of CONSORT for Abstracts items, whereas articles published in ENT journals report a mean of 32.3% (26.6–38.0%; median 31.6%) (p < 0.001).

**Fig 3:**
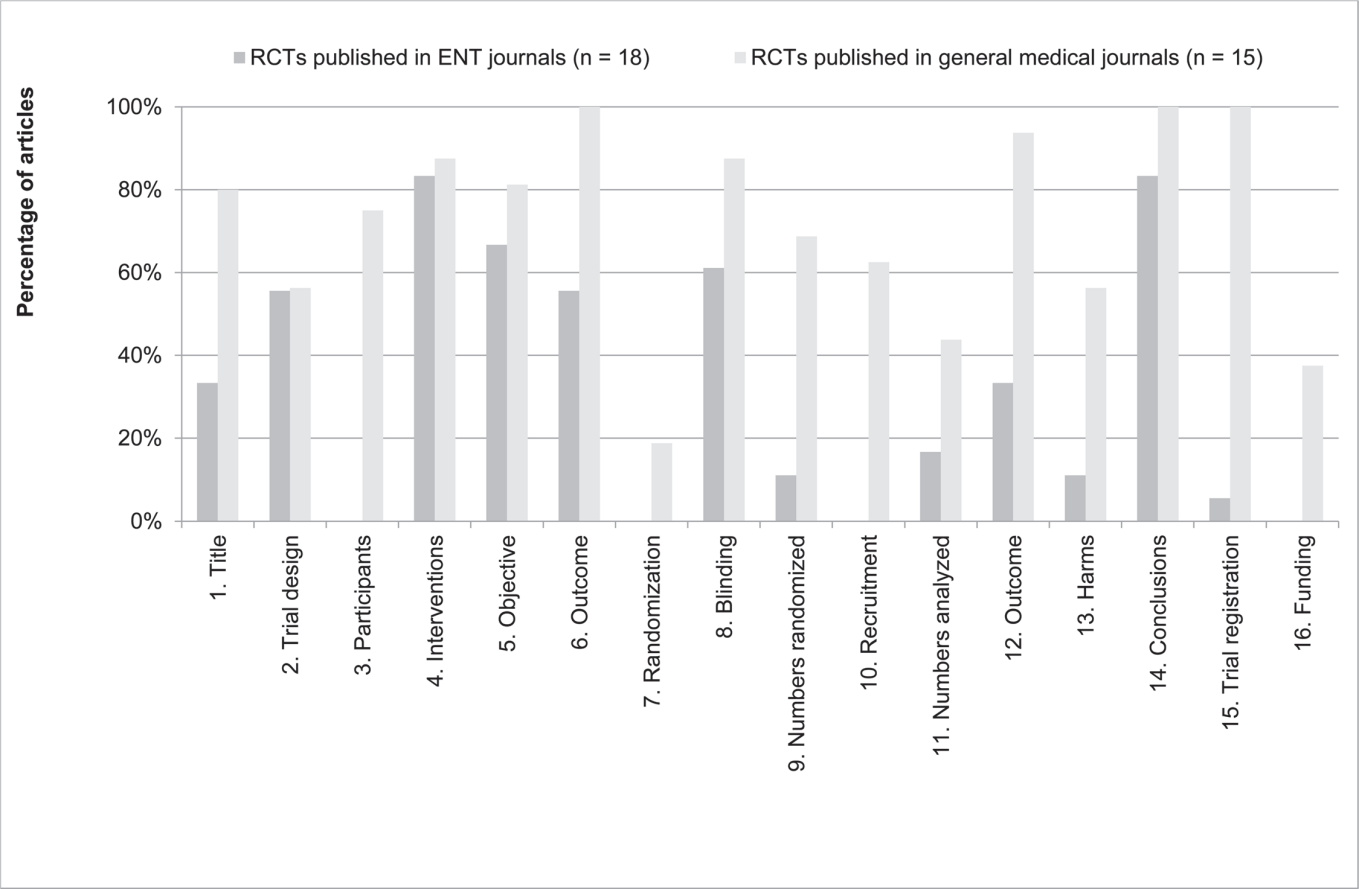
Reporting of CONSORT for Abstracts items per journal type. The percentage of articles reporting CONSORT for Abstract items adequately (in %), sorted per journal type (articles published in general medical journals, n = 15; articles published in ENT journals, n = 18). On the original CONSORT for Abstracts checklist [12], item 2 (author names) is specific for conference abstracts only. Therefore, we renumbered the subsequent items.

When taking a closer look on individual CONSORT for Abstracts items, again there are large differences (Fig. 3). First, no abstract of an article published in ENT journals adequately described Participants (item 3). Second, both abstracts of articles from ENT journals and general medical journals report Randomisation (item 7) inadequately (0 and 19%, respectively). Finally, Recruitment, Numbers analysed, Harms, Trial registration and Funding were adequately reported in <20% of all articles published in ENT journals.

The abstracts of articles published in general medical journals had a mean of 337.7 ± 35.7 [267–405] words, whereas abstracts of articles published in ENT journals had a mean of 204.3 ± 69.1 [84–331] words. This difference was statistically significant (p < 0.001). Furthermore, there was a significant correlation between abstract word count and number of adequately reported CONSORT for Abstracts items (Spearman’s rho *p* = 0.01, correlation coefficient 0.736, sig (2-tailed) 0.000).

## Discussion

To our knowledge, this is the first study evaluating the quality of reports and abstracts of RCTs in otorhinolaryngologic literature. We evaluated the quality of reporting using the CONSORT Statement checklists [10, 12]. We compared articles published in general medical journals with articles published in ENT journals and found adequate reporting of CONSORT items in 92.1% (89.5–94.7%) and 71.8% (66.7–76.8%) respectively (p < 0.001). Large differences in the quality of reporting RCTs were observed between both journal types.

There are several topics essential to RCTs that were reported inadequately (Fig. 2). First, 72% of articles published in ENT journals fail to report details of the sample size calculation (versus 0% in general medical journals). Also in other areas of expertise, less than half of the articles describe sample size calculations correctly [1, 17, 20, 21]. The randomisation procedure is considered of utmost importance in RCTs. Yet articles do not report this process sufficiently. CONSORT items Sequence generation, Allocation concealment mechanism, Implementation are inadequately reported in >50% of articles published in ENT journals. This figure is again congruent with other medical specialties [1, 3, 21–23] and a recent Cochrane review on the completeness of reporting RCTs [17]. Studies published in ENT journals were not registered or did not mention trial registration (89%) in a trial register and none of the articles had a previously published study protocol, yielding a risk of bias by selective reporting [4, 6]. Clinical trial registration before patient enrolment is part of the Declaration of Helsinki and is required by the International Committee of Medical Journal Editors (ICMJE) [24]. The articles published in general medical journals score better on all these items. It has been highlighted before that reporting of essential items is better in general medical journals; it is thought to result from more stringent requirements by the editorial offices of the general medical journals [7].

In the analysis of reporting CONSORT for Abstracts items again a large difference between articles published in general medical journals and ENT journals can be observed (Fig. 3). A significant correlation between the number of words and correctly reporting CONSORT for Abstract items was observed (Spearman’s rho, p = 0.01), suggesting that authors simply need a sufficient amount of words to correctly report all necessary study information in their abstracts. In Table 4, the maximum number of abstract words is sorted for the top 5 general medical and ENT journals, showing large differences between these types.

**Table 4:** Maximum number of words in abstracts and endorsement of CONSORT Statement, per journal.

Journal	Maximum number of words	Endorse CONSORT?
General medical journals		
1. NEJM	250	Yes
2. Lancet	300	Yes
3. JAMA	350	Yes
4. BMJ	*No fixed limit*, *‘to encourage full reporting’*	Yes
5. PLOS Med	300	Yes
ENT journals		
1. Ear Hear	500	No
2. JARO	250	No
3. Head Neck	150	No
4. Hear Res	NA	No
5. Audiol Neurotol	*‘10 lines’*	No

Source: Instructions to Authors section on journals’ websites, accessed July 17^th^, 2014. NA = not available.

Table 4 also shows that all general medical journals have endorsed the CONSORT Statement, meaning that these journals refer to the CONSORT Statement in the Instructions to Authors section on their websites (NEJM, Lancet) or even request a completed checklist to be uploaded with the submission of their manuscript (BMJ, JAMA, PLOS Med; personal communication of first author with editors, July 2014). None of the ENT journals report the CONSORT Statement in the Instructions to Authors section on their website (date of access July 8^th^, 2014), even though there are more than 200 medical journals that endorse CONSORT worldwide [25, 26].

Strengths of our study include our transparent search strategy to retrieve all RCTs. It can be easily reproduced, since all complete syntaxes are provided or referred to (Fig. 1, S1 File). Next, we were very thorough in our analysis and for instance contacted editorial offices for further information. We gave an overview of all CONSORT items and did not select a few CONSORT items that we considered most important. If possible, we used an appropriate CONSORT extension to assess individual articles or items, e.g. non-pharmacologic treatments, CONSORT for Abstracts or Harms [12–14]. Furthermore, we compare our data with other (surgical) medical specialties to put our findings in a broader perspective. Finally, we transformed our results into practical recommendations for authors and editors (see Recommendations).

Our study also has some limitations. First, interpreting phrases in articles and assessing them on a qualitative scale will always remain subjective. We think we countered this sufficiently by ensuring that two independent reviewers assessed whether articles were RCTs and conducted in the otorhinolaryngologic field and by making our scoring system publicly available (Appendices 2 and 3). Another limitation could be that we performed our search only in the Pubmed database, excluding other medical databases. However, Pubmed is one of the largest and most widely available databases comprising over 24 million citations [27]. Furthermore, we used the CONSORT 2010 checklist to assess the quality of included articles. One may argue that authors of articles published in 2010 had no chance to report according to this version of the CONSORT Statement. Nonetheless, previous versions of the CONSORT Statement were long available to help authors report adequately [8, 9]. Ultimately, reporting according to the CONSORT Statement does not necessarily result in better reporting of studies. For example, the three studies that report according to the CONSORT Statement that were published in general medical journals report a mean of 98.2% (96.4–100%) of CONSORT items adequately. This is, however, a higher mean score than the score of the articles not reporting according to the CONSORT Statement published in general medical journals 90.5% (88.0%–93.1%).

### Recommendations

Correct reporting of clinical trials is an important aspect of good research and essential for clinicians and researchers to value the results of trials. To assist adequate reporting, the CONSORT Statement can be a helpful tool. We advise authors in the otorhinolaryngologic field to use the CONSORT Statement when reporting an RCT. It has been shown that reporting according to the CONSORT Statement has a beneficial influence on the quality of reporting [15–17]. We recommend editors of ENT journals to endorse the CONSORT Statement in the Instructions for Authors section on their website and to make it a requirement that a CONSORT checklist is submitted along with a new manuscript. Finally, a larger maximum number of words should be allowed for abstracts of RCTs to assure accurate description of all essential trial information.

## Conclusion

The quality of reports of RCTs in otorhinolaryngologic literature assessed with the CONSORT checklists is suboptimal. This results in risk of bias, and therefore the value of these trials for clinicians and researchers is limited. RCTs published in general medical journals (that all endorse the CONSORT Statement) have a higher quality of reporting than articles published in ENT journals. We advise authors to report their trial according to the CONSORT Statement. Finally, we recommend editors to endorse the CONSORT Statement and to require the submission of CONSORT checklist along with a new manuscript prior to peer review to ensure more adequate reporting of RCTs.

## Supporting Information

S1 File Complete search syntax. a) The editorial team, Cochrane Ear Nose and Throat Disorders Group. About the Cochrane Collaboration (Cochrane Reviews Group (CRGs)), 2012 issue 7, art. no.: ENT. CENTRAL search strategy. Available: http://onlinelibrary.wiley.com/o/cochrane/clabout/articles/ENT/sect0-meta.html. Accessed on 25 June 2014. b) The Cochrane Collaboration (March 2011). Cochrane Highly Sensitive Search Strategy for identifying randomized trials in MEDLINE: sensitivity-maximizing version (2008 revision); PubMed format. Cochrane Handbook for Systematic Reviews for Interventions, Box 6.4.a. Available handbook.cochrane.org. Accessed 25 June 2014.
(DOC)

S2 File Explanation of criteria to score as ‘adequately reported CONSORT item’. Please see Schulz et al. [10] for the original CONSORT 2010 checklist and Moher et al. [11] for explanation and elaboration including scoring guidelines. When the trial evaluated non-pharmacologic treatments, we assessed the articles using additions from Boutron et al. [14]. * Marked items concern optional items. When possible in the study and adequately reported, the item was scored as ‘adequately reported’. When possible, but not reported, the item was scored as ‘inadequately reported’. If not possible, the item was not scored as ‘inadequately reported’, but left open.
(DOCX)

S3 File Explanation of criteria to score as ‘adequately reported CONSORT for Abstract item’. On the original CONSORT for Abstracts checklist [12], item 2 is Author Information. Since this item is specific for conference abstracts only, we omitted this item and subsequently number the other items. * Marked items concern optional items. When possible in the study and reported, the item was scored as adequately reported. When possible, but not reported, the item was scored as inadequately reported. When not possible, the item was not scored as inadequately reported.
(DOCX)

## References

[1] Agha R, Cooper D, Muir G. The reporting quality of randomised controlled trials in surgery: a systematic review. Int J Surg 2007;5(6): 413–22. 18029237 10.1016/j.ijsu.2007.06.002

[2] Jüni P, Altman DG, Egger M. Systematic reviews in health care: assessing the quality of controlled clinical trials. BMJ 2001;323: 42–46. 11440947 10.1136/bmj.323.7303.42 PMC1120670

[3] Chan AW, Altman DG. Epidemiology and reporting of randomised trials published in PubMed journals. Lancet 2005;365(9465): 1159–62. 15794971 10.1016/S0140-6736(05)71879-1

[4] Van de Wetering FT, Scholten RJ, Haring T, Clarke M, Hooft L. Trial registration numbers are underreported in biomedical publications. PLOS One 2012;7(11): e49599 10.1371/journal.pone.0049599 23166724 PMC3498197

[5] Chan AW, Hróbjartsson A, Haahr MT, Gøtzsche PC, Altman DG. Empirical evidence for selective reporting of outcomes in randomized trials. JAMA 2004;291(20): 2457–65. 15161896 10.1001/jama.291.20.2457

[6] Hewitt C, Hahn S, Torgerson DJ, Watson J, Bland JM. Adequacy and reporting of allocation concealment: review of recent trials published in four general medical journals. BMJ 2005;30(7499): 1057–8. 15760970 10.1136/bmj.38413.576713.AE PMC557225

[7] Mills EJ, Wu P, Gagnier J, Devereaux PJ. The quality of randomized trial reporting in leading medical journals since the revised CONSORT statement. Contemp Clin Trials 2005;26(4): 480–7. 16054580 10.1016/j.cct.2005.02.008

[8] Begg C, Cho M, Eastwood S, Horton R, Mohr D, Olkin I, et al Improving the quality of reporting of randomized controlled trials. The CONSORT Statement. JAMA 1996;276: 637–9. 8773637

[9] Moher D, Schulz KF, Altman DG. The CONSORT Statement: revised recommendations for improving the quality of reports of parallel-group randomized trials. Lancet 2001;357(9263): 1191–1194. 11323066

[10] Schulz KF, Altman DG, Moher D for the CONSORT group. CONSORT 2010 statement: updated guidelines for reporting parallel group randomised trials. BMJ 2010;340: c332 10.1136/bmj.c332 20332509 PMC2844940

[11] Moher D, Hopewell S, Schulz KF, Montori V, Gøtzsche PC, Devereaux PJ, et al for the CONSORT group. CONSORT 2010 explanation and elaboration: updated guidelines for reporting parallel groups randomised trials. BMJ 2010;340: c869 10.1136/bmj.c869 20332511 PMC2844943

[12] Hopewell S, Clarke M, Moher D, Wager E, Middleton P, Altman DG, et al for the CONSORT group. CONSORT for reporting randomized controlled trials in journal and conference abstracts: explanation and elaboration. PLOS Med 2008;5(1): e20 10.1371/journal.pmed.0050020 18215107 PMC2211558

[13] Ioannidis JP, Evans SJ, Gøtzsche PC, O’Neill RT, Altman DG, Schulz K, et al for the CONSORT group. Better reporting of harms in randomized trials: an extension of the CONSORT Statement. Ann Intern Med 2004;141(10): 781–788. 15545678 10.7326/0003-4819-141-10-200411160-00009

[14] Boutron I, Moher D, Altman DG, Schulz K, Ravaud P, for the CONSORT group. Extending the CONSORT Statement to randomized trials of nonpharmacologic treatment: an explanation and elaboration. Ann Intern Med 2008;148: 295–309. 18283207 10.7326/0003-4819-148-4-200802190-00008

[15] Moher D, Jones A, Lepage L, for the CONSORT group. Use of the CONSORT statement and quality of reports of randomized trials: a comparative before-and-after evaluation. JAMA 2001;285(15): 1992–5. 11308436 10.1001/jama.285.15.1992

[16] Plint AC, Moher D, Morrison A, Schulz K, Altman DG, Hill C, et al Does the CONSORT checklist improve the quality of reports of randomised controlled trials? A systematic review. Med J Aust 2006;185(5): 263–7. 16948622 10.5694/j.1326-5377.2006.tb00557.x

[17] Turner L, Shamseer L, Altman DG, Weeks L, Peters J, Kober T, et al Consolidated standards of reporting trials (CONSORT) and the completeness of reporting randomized controlled trials (RCTs) published in medical journals. Cochrane Database Syst Rev 2012;11: MR000030 10.1002/14651858.MR000030.pub2 23152285 PMC7386818

[18] The editorial team, Cochrane Ear Nose and Throat Disorders Group. About the Cochrane Collaboration (Cochrane Reviews Group (CRGs)), 2012 issue 7, art. no.: ENT. CENTRAL search strategy. Available: http://onlinelibrary.wiley.com/o/cochrane/clabout/articles/ENT/sect0-meta.html. Accessed 2014 Jun 25.

[19] The Cochrane Collaboration (March 2011). Cochrane Highly Sensitive Search Strategy for identifying randomized trials in MEDLINE: sensitivity-maximizing version (2008 revision); PubMed format. Cochrane Handbook for Systematic Reviews for Interventions, Box 6.4.a. Available handbook.cochrane.org. Accessed 2014 Jun 25.

[20] Lai TY, Wong VW, Lam RF, Cheng AC, Lam DS, Leung GM. Quality of reporting of key methodological items of randomized controlled trials in clinical ophthalmic journals. Ophthalmic Epidemiol 2007;14(6): 390–8. 18161613 10.1080/09286580701344399

[21] Rios LP, Odueyungbo A, Moitri MO, Rahman, Thabane L. Quality of reporting randomized controlled trials in general endocrinology literature. J Clin Endocrinol Metab 2008;93(10): 3810–6. 10.1210/jc.2008-0817 18583463

[22] Agha RA, Camm CF, Edison E, Orgill DP. The methodological quality of randomized controlled trials in plastic surgery needs improvement: a systematic review. J Plast Reconstr Aesthet Surg 2013;66(4): 447–52. 10.1016/j.bjps.2012.11.005 23245758

[23] Balasubramanian SP, Wiener M, Alshameeri Z, Tiruvoipati, Elbourne D, Reed MW. Standards of reporting randomized controlled trials in general surgery: can we do better? Ann Surg 2006;244(5): 663–7. 17060756 10.1097/01.sla.0000217640.11224.05 PMC1856614

[24] International Committee of Medical Journal Editors (September 2004). Clinical Trial Registration, News & Editorials. Available: http://www.icmje.org/news-and-editorials/clin_trial_sep2004.pdf. Accessed 2014 Jul 29.

[25] Altman DG. Endorsement of the CONSORT Statement by high impact medical journals: survey of instructions for authors. BMJ 2005;330: 1056–7. 15879389 10.1136/bmj.330.7499.1056 PMC557224

[26] Hopewell S, Altman DG, Moher D, Schulz KF. Endorsement of the CONSORT Statement by high impact factor medical journals: a survey of journal editors and journal ‘Instructions to Authors’. Trials 2008;9: 20 10.1186/1745-6215-9-20 18423021 PMC2359733

[27] National Institutes of Health, National Library of Medicine (9 July 2014). Pubmed Help. http://www.ncbi.nlm.nih.gov/books/NBK3827/. Accessed 2014 Jul 29.

