# Supplementary material for: Assessment of the Quality of Reporting of Randomised Controlled Trials in Otorhinolaryngologic Literature – Adherence to the CONSORT Statement
Source: PLoS One. 2015 Mar 20;10(3):e0122328. doi: 10.1371/journal.pone.0122328 (PMC4368673; doi:10.1371/journal.pone.0122328)
Supplement: S1 File — a) The editorial team, Cochrane Ear Nose and Throat Disorders Group. About the Cochrane Collaboration (Cochrane Reviews Group (CRGs)), 2012 issue 7, art. no.: ENT. CENTRAL search strategy. Available: http://onlinelibrary.wiley.com/o/cochrane/clabout/articles/ENT/sect0-meta.html. Accessed on 25 June 2014. b) The Cochrane Collaboration (March 2011). Cochrane Highly Sensitive Search Strategy for identifying randomized trials in MEDLINE: sensitivity-maximizing version (2008 revision); PubMed format. Cochrane Handbook for Systematic Reviews for Interventions, Box 6.4.a. Available handbook.cochrane.org. Accessed 25 June 2014. (DOC) [file pone.0122328.s001.doc]

**Supporting Information 1**

| **Search** | **Syntax** | | **Result** |
| --- | --- | --- | --- |
| #1 | (((randomized controlled trial[pt]) OR (controlled clinical trial[pt]) OR (randomized[tiab]) OR (placebo[tiab]) OR (clinical trials as topic [mh:noexp]) OR (randomly[tiab]) OR (trial[ti])) AND humans[mh])) ^a^ | | 761,845 |
| #2 | **Search no.** | **ENT subject CENTRAL** ^b^ | 469,905 |
|  | #1 | Otorhinolaryngologic diseases[MeSH Terms] |  |
|  | #2 | Otorhinolaryngologic neoplasms[MeSH Terms] |  |
|  | #3 | Otorhinolaryngologic surgical procedures[MeSH Terms] |  |
|  | #4 | (mastoid*[ti] OR ossic*[ti] OR stape*[ti] OR cochlea*[ti] OR auric*[ti] OR labyrinth*[ti] OR auditory[ti] OR hearing[ti] OR ((auditory[ti] OR acoustic[ti]) AND nerve[ti]) OR vestibul*[ti] OR eardrum*[ti] OR tympan*[ti] OR "eustachian tube"[ti] OR ramsay[ti] OR (towne*[ti] AND brock[ti]) OR branchio*[ti] OR "cochleo vestibular"[ti] OR cochleovestibular[ti] OR pinna*[ti] OR acoustic[ti] OR maxillary[ti] OR jaw[ti] OR mandibular[ti] OR mandible[ti]) |  |
|  | #5 | (neck[ti] OR nose[ti] OR nasal[ti] OR endonasal[ti] OR paranasal[ti] OR olfact*[ti] OR turbinat*[ti] OR choana*[ti] OR antrochoana*[ti] OR adenoid*[ti] OR postcricoid*[ti] OR intranasal[ti] OR sinus*[ti] OR smell*[ti] OR cilia*[ti] OR septal[ti] OR saliva*[ti] OR parotid[ti] OR sublingual[ti] OR submandibular[ti] OR submaxillary[ti] OR sicca[ti] OR stensen*[ti] OR (wharton*[ti] AND (gland* OR duct*))) |  |
|  | #6 | (pharyn*[ti] OR nasopharyn*[ti] OR oropharyn*[ti] OR hypopharyn*[ti] OR retropharyn*[ti] OR rhinopharyn*[ti] OR parapharyn*[ti] OR palatopharyn*[ti] OR velopharyn*[ti] OR palatal[ti] OR palatine[ti] OR velopalatine[ti] OR throat[ti] OR tonsil*[ti] OR retrotonsil*[ti] OR peritonsillar[ti] OR laryn*[ti] OR voice[ti] OR "vocal cord"[ti] OR “vocal cords”[ti] OR "vocal fold"[ti] OR “vocal folds”[ti] OR glottis[ti] OR epiglot*[ti] OR supraglot*[ti] OR epilaryn*[ti] OR lemierre[ti]) |  |
|  | #7 | #4 OR #5 OR #6 |  |
|  | #8 | (disease*[ti] OR disorder*[ti]) |  |
|  | #9 | (infect*[ti] OR inflamm*[ti] OR pain*[ti] OR surg*[ti] OR neoplasm*[ti] OR cancer*[ti] OR tumour*[ti] OR tumor*[ti] OR carcinom*[ti] OR treatment*[ti] OR therap*[ti] OR malignan*[ti]) |  |
|  | #10 | #8 OR #9 |  |
|  | #11 | #7 AND #10 |  |
|  | #12 | (#1 OR #2 OR #3 OR #11) |  |
|  | #13 | (oticus[ti] OR otoliq*[ti] OR ototox*[ti] OR otolog*[ti] OR otoplasty[ti] OR pinnaplasty[ti] OR ossiculoplasty[ti] OR staped*[ti] OR "ear wax"[ti] OR earwax[ti] OR cerumen[ti] OR "ear effusion"[ti] OR “ear effusions”[ti] OR "glue ear"[ti] OR otosclerosis[ti] OR otospongiosis[ti] OR paracusis[ti] OR presbyacusis[ti] OR "auditory nerve"[ti] OR “auditory nerves”[ti] OR deaf[ti] OR deafness[ti] OR hyperacusis[ti] OR hypoacusis[ti] OR "hearing impaired"[ti] OR "hearing loss"[ti] OR "loudness recruitment"[ti] OR "loudness perception"[ti] OR "auditory inattention"[ti]) |  |
|  | #14 | (alport[ti] OR aldrich[ti] OR brock[ti] OR gradenigo*[ti] OR "herpes zoster oticus"[ti] OR cephalicus[ti] OR "perilymph fistula"[ti] OR "perilymph fistulas"[ti] OR "cochlear hydrops"[ti] OR "endolymphatic hydrops"[ti] OR (hydrops[ti] AND labyrinth*[ti]) OR ((motion[ti] OR air[ti] OR car[ti] OR travel[ti] OR sea[ti]) AND (sick*[ti])) OR seasick*[ti] OR carsick*[ti] OR dizziness[ti]) |  |
|  | #15 | (earplug*[ti] OR (ear[ti] AND (plug*[ti] OR mold*[ti] OR mould*[ti])) OR (hearing[ti] AND (aid*[ti] OR device*[ti])) OR ((hearing[ti] OR vestibular[ti]) AND rehabilitation[ti]) OR "endolymphatic shunt"[ti] OR "labyrinth fenestration" OR “ear inflation”[ti] OR autoinflation[ti] OR ventilation[ti] OR (tympanostomy[ti] AND tube*[ti]) OR grommet*[ti] OR myringostomy[ti] OR myringotomy[ti] OR myringoplasty[ti] OR epley[ti]) |  |
|  | #16 | (((hearing[ti] OR vestibular[ti] OR caloric[ti] OR barany*[ti] OR “acoustic impedence”[ti]) AND test*[ti]) OR "dichotic listening"[ti] OR audiometr*[ti] OR audiography[ti] OR audiology[ti] OR otoscopy[ti] OR tonotopy[ti] OR electronystagmography[ti] OR cochleostomy[ti] OR mastoidectomy[ti] OR ((auditory[ti] OR ear[ti] OR cochlea*[ti]) AND (implant[ti] OR implants[ti] OR implantation*[ti]))) |  |
|  | #17 | ("nasal provocation"[ti] OR "nose provocation"[ti] OR epistax*[ti] OR nosebleed*[ti] OR ((nose[ti] OR nasal[ti) AND bleed*[ti]) OR (nasal[ti] AND liquorr*[ti]) OR anosmi*[ti] OR cacosmi*[ti] OR dysosmi*[ti] OR hyposmi*[ti] OR paraosmi*[ti] OR (anteverted[ti] AND (nares[ti] OR nostril*[ti])) OR antritis[ti] OR rhinitis[ti] OR pollinosis[ti] OR pollenosis[ti] OR hayfever[ti] OR “hay fever”[ti] OR rhinolog*[ti] OR rhinoman*[ti] OR rhinosinusitis[ti] OR rhinoconjunctivit*[ti]) |  |
|  | #18 | (rhinolaryn*[ti] OR rhinopharyn*[ti] OR rhinoscler*[ti] OR rhinoscop*[ti] OR rhinosept*[ti] OR adenoiditis[ti] OR hypernasality[ti] OR hyponasality[ti] OR ((cleft[ti] AND lip[ti]) AND (nose[ti] OR nasal[ti] OR nostril[ti])) OR sinusitis[ti] OR parasinusitis[ti] OR snoring[ti] OR snore[ti] OR sneez*[ti] OR esthesioneuroblastoma[ti] OR "nasal glioma"[ti] OR "nasal heterotopia"[ti] OR (choan*[ti] AND (atresia[ti] OR stenosis[ti]))) |  |
|  | #19 | (((nose[ti] OR nasal[ti] OR sinus[ti]) AND (scleroma[ti] OR mucocele[ti])) OR dacryocystorhinostomy[ti] OR septoplasty[ti] OR septorhinoplasty[ti] OR adenoidectomy[ti] OR ((nasal[ti] OR nose[ti]) AND (pack*[ti] OR tampon*[ti] OR decongest*[ti] OR drops[ti]))) |  |
|  | #20 | (((pharyn*[ti] OR laryn*[ti] OR oesophageal[ti] OR esophageal[ti]) AND reflux[ti]) OR pharyngectomy[ti] OR pharyngostomy[ti] OR tonsillectomy[ti] OR tonsillotomy[ti] OR pharyngoplasty[ti] OR velopharyngoplasty[ti] OR nasopharyn*[ti] OR pharyngitis[ti] OR quinsy[ti] OR "peritonsillar abscess"[ti] OR tonsillitis[ti] OR tonsilitis[ti] OR "globus pharyngeus"[ti] OR "sore throat"[ti] OR epipharyngitis[ti] OR laryngitis[ti] OR uvulopalatopharyngoplasty[ti] OR uvulopharyngopalatoplasty[ti] OR uppp[ti]) |  |
|  | #21 | (((pharyn*[ti] OR zenker*[ti] OR esophagopharyn*[ti]) AND (divert* OR pouch*[ti])) OR laryngismus[ti] OR laryngomalacia[ti] OR laryngitis[ti] OR (laryn*[ti] AND (malacia[ti] OR web[ti] OR dystonia[ti] OR speech[ti] OR granuloma[ti])) OR dysphoni*[ti] OR aphoni*[ti] OR hoarse*[ti] OR laryngocele[ti] OR laryngostenosis[ti] OR laryngotrach*[ti] OR laryngospasm*[ti]) |  |
|  | #22 | (”upper respiratory tract infection"[ti] OR (upper[ti] AND (airway*[ti] OR respiratory[ti]) AND infection*[ti]) OR (urt[ti] AND (inflamm*[ti] OR infect*[ti])) OR urti[ti] OR parotitis[ti] OR parotiditis[ti] OR sialadenitis[ti] OR sialodenitis[ti] OR sialitis[ti] OR asialia[ti] OR hyposalivation[ti] OR hypersalivation[ti] OR hyposialorrh*[ti] OR hypersialorrh*[ti] OR ptyalism[ti] OR sialosis[ti] OR sialometaplasia[ti] OR branchiom*[ti] OR "glomus caroticum"[ti] OR "carotid paraganglioma"[ti]) |  |
|  | #23 | (#13 OR #14 OR #15 OR #16 OR #17 OR #18 OR #19 OR #20 OR #21 OR #22) |  |
|  | #24 | (#12 OR #23 |  |
| #3 | (“2010”[Date – Publication] : “3000”[Date – Publication]) | | 4,218,013 |
| #4 | (("N Engl J Med"[jour] OR "Lancet"[jour] OR "JAMA"[jour] OR “BMJ”[jour] OR "PLoS Med"[jour])) | | 328,241 |
| #5 | "Ear Hear"[jour] OR "J Assoc Res Otolaryngol"[jour] OR "Head Neck"[jour] OR "Hear Res"[jour] OR "Audiol Neurootol"[jour] | | 13,658 |
| #6 | #1 AND #2 AND #3 AND #4 | | 40 |
| #7 | #1 AND #2 AND #3 AND #5 | | 69 |
