# Supplementary material for: Assessment of the Quality of Reporting of Randomised Controlled Trials in Otorhinolaryngologic Literature – Adherence to the CONSORT Statement
Source: PLoS One. 2015 Mar 20;10(3):e0122328. doi: 10.1371/journal.pone.0122328 (PMC4368673; doi:10.1371/journal.pone.0122328)
Supplement: S2 File — Please see Schulz et al. [10] for the original CONSORT 2010 checklist and Moher et al. [11] for explanation and elaboration including scoring guidelines. When the trial evaluated non-pharmacologic treatments, we assessed the articles using additions from Boutron et al. [14]. * Marked items concern optional items. When possible in the study and adequately reported, the item was scored as ‘adequately reported’. When possible, but not reported, the item was scored as ‘inadequately reported’. If not possible, the item was not scored as ‘inadequately reported’, but left open. (DOCX) [file pone.0122328.s002.docx]

**Supporting Information 2**

| **Item** | | **Criteria to score as adequately reported** |
| --- | --- | --- |
| *Title and Abstract* | | |
| 1a | Title | The title of the manuscript contained a word with random* in its root. |
| 1b | Abstract | See *Supporting Information 3* |
| *Introduction* | | |
| 2a | Background | Background information was provided. We did not judge the quality of the background information. |
| 2b | Objective/  Hypothesis | A hypothesis OR objective was mentioned. |
| *Methods* | | |
| 3a | Trial design | The trial design was described (parallel, cluster RCT etc.) or sufficient data was provided about the trial design, including the allocation ratio. |
| 3b* | Changes to methods | When applicable, important changes to methods were described, with reasons for changes. We could not know if all changes to methods were reported. |
| 4a | Eligibility criteria | Both in- AND exclusion criteria were provided. |
| 4b | Settings and locations | The settings where the data were collected were described. |
| 5 | Interventions | Interventions were described in sufficient detail to allow replication at other centers. Surgical procedures needed to be outlined. |
| 6a | Primary/secondary outcomes | A primary outcome was defined, with optional secondary outcomes. |
| 6b* | Changes to outcomes | When applicable, important changes to outcomes were described, with reasons for changes. We could not know if all changes to outcomes were reported. |
| 7a | Sample size | A statistical calculation for the sample size was provided. |
| 7b* | Interim analysis, stopping guidelines | When applicable, interim analyses and stopping guidelines were described. |
| 8a | Method sequence generation | The method of sequence generation was described, e.g. computer-based. |
| 8b | Type of randomisation | The type of randomisation was described, e.g. minimisation or block. Unless stated, we could not be sure if any restrictions had been used. |
| 9 | Allocation concealment | Mechanism to conceal allocation were described. |
| 10 | Implementation | It was described who generated the allocation sequence, enrolled participants and assigned participants. |
| 11a* | Blinding | When possible, blinding of interventions described (participants, care professionals, research staff). If not possible, not scored as inadequate. |
| 11b* | Similarity of interventions | When relevant, the similarity of interventions was described. For example, placebo looked like investigational product; not relevant when comparing surgical technique vs. no surgery. |
| 12a | Statistical methods | The statistical procedures to analyse the data were provided. |
| 12b* | Additional analyses | When relevant, additional statistical procedures were described. |
| *Results* | | |
| 13a | Participants | Numbers of participants randomly assigned to groups, received intended treatment and analysed for primary outcome were described. Although a flow diagram is highly recommended, it was not required. |
| 13b | Losses and exclusions | Losses and exclusions for all groups were described, with reasons. |
| 14a | Recruitment | The dates of recruitment and follow up were noted. |
| 14b | End of trial | A reason for ending the trial was given. When the sample size was reached, we considered this to be the reason for ending the trial. Otherwise, specific reasons needed to be stated. |
| 15 | Baseline data | Baseline group characteristics shown (table required). |
| 16 | Numbers analysed | Number of patients analysed per outcome was described. |
| 17a | Primary/secondary outcomes | Results per group for primary and, optionally, secondary outcomes, together with effect size or precision. Only p-values reported was not considered to be adequately reported. |
| 17b* | Binary outcomes | When applicable, both absolute and relative effect sizes for binary outcomes. |
| 18* | Ancillary analyses | When relevant, additional analyses performed were described. |
| 19 | Harms | Harms or adverse events described for all groups [13]. When no adverse events occurred, this also had to be stated. |
| *Discussion* | | |
| 20 | Limitations | Possible sources of bias, imprecision and study weaknesses must be discussed. |
| 21 | Generalisability | An attempt of the authors to generalise their study results and discuss applicability and relevance in future was described. |
| 22 | Interpretation | An interpretation of the trial results, benefits and harms, and considering other evidence was provided. |
| *Other information* | | |
| 23 | Registration | Name of trial registry and trial registration number are provided. Not scored if trial was only registered at local ethics committee. |
| 24 | Protocol | A statement of where the study protocol could be retrieved was provided. We think all RCTs (should) have a protocol available, so scored inadequate if not provided. |
| 25 | Funding | Sources of support and role of funders was mentioned. |
