# Supplementary material for: Assessment of the Quality of Reporting of Randomised Controlled Trials in Otorhinolaryngologic Literature – Adherence to the CONSORT Statement
Source: PLoS One. 2015 Mar 20;10(3):e0122328. doi: 10.1371/journal.pone.0122328 (PMC4368673; doi:10.1371/journal.pone.0122328)
Supplement: S3 File — On the original CONSORT for Abstracts checklist [12], item 2 is Author Information. Since this item is specific for conference abstracts only, we omitted this item and subsequently number the other items. * Marked items concern optional items. When possible in the study and reported, the item was scored as adequately reported. When possible, but not reported, the item was scored as inadequately reported. When not possible, the item was not scored as inadequately reported. (DOCX) [file pone.0122328.s003.docx]

**Supporting Information 3**

| **Item** | | **Criteria to score as adequately reported** |
| --- | --- | --- |
| 1 | Title | The title of the manuscript contained a word with random* in its root. |
| 2 | Trial design | The trial design was described using specific words (e.g. non-inferiority, cluster, parallel, etc.) |
| *Methods* | | |
| 3 | Participants | Both in- AND exclusion criteria, AND settings where the data were collected are stated. |
| 4 | Interventions | The interventions were described. |
| 5 | Objective | Objectives OR hypotheses was stated. |
| 6 | Outcome | The primary outcome was mentioned. |
| 7 | Randomisation | The allocation of participants to interventions was described. |
| 8* | Blinding | When applicable, details of blinding were provided. |
| *Results* | | |
| 9 | Numbers randomised | Numbers randomised to each group are described. |
| 10 | Recruitment | Dates of recruitment and follow up period stated. |
| 11 | Numbers analysed | Numbers analysed in each group are described. |
| 12 | Outcome | For the primary outcome, group results are provided with effect size or precision. Only p-values reported was not considered to be adequately reported. |
| 13 | Harms | Harms or adverse events described for all groups. When no adverse events occurred, this must also be stated. |
| 14 | Conclusions | General interpretation of the results must be described. |
| 15 | Trial registration | Name of trial registry and trial registration number are provided. |
| 16 | Funding | Sources of support or funding were mentioned. |
